# Displaced lives: rethinking survival, social reproduction, and (in)security with refugees

**DOI:** 10.1080/14616742.2025.2456595

**Published:** 2025-02-12

**Authors:** Raksha Gopal, Luisa Lupo

**Affiliations:** International Relations/Political Science, Geneva Graduate Institute, Geneva, Switzerland

**Keywords:** Forced displacement, survival, social reproduction, feminist political economy, security

## Abstract

In this article, we examine the practices of survival that Rohingya and Syrian refugees perform as they confront multiple forms of violence resulting from their forced displacement in India and Turkey, respectively. We consider these practices as they are performed in the everyday and reflect on how they expand existing debates in social reproduction feminism. Social reproduction refers to those practices that are essential for the everyday and generational maintenance of life. First, we show that for people living in conditions of prolonged displacement and violence, practices of social reproduction become a matter of survival that entails “making secure” amid the insecurity of displacement. Second, we demonstrate that these practices highlight the role of not only the welfare state but also the security state for social reproduction. We propose the concept of the “(in)securitization of social reproductive capacities” to examine how state and non-state actors hinder social reproduction as much as they support it and how displaced people negotiate with this. We conclude that survival, including the ways in which refugees cope with insecurity, care, and sustain their lives, can be a meaningful tool to pluralize understandings of social reproduction, bridging insights from feminist political economy, critical migration, and security studies.

## Introduction

Amal^1^ tells me (Luisa) and Samira (my interpreter, also a refugee from Syria) about her experience of displacement in Turkey, loss, and survival as a result of the war in Syria. “I never thought I would come and live here if it weren’t for the war,”^2^ she says, as we drink tea sitting on embroidered cushions in the place where she currently lives with her family, in a village in Şanlıurfa, southeast Turkey, not far from the Turkey–Syria border. She confides that she feels possessed by an evil force that prevents her from performing daily tasks such as cooking, cleaning, and taking care of her children: “[I]t’s like I’m here but not here.” This feeling of suspension is the result of the traumatic effects of the war and losing loved ones. At the same time, she says that it is those very mundane tasks that keep her and her family grounded, make her children feel loved, and create a sense of resilience and fulfillment.^3^

Meanwhile, 3,000 kilometers away in a *jhuggi* (slum dwelling made of tarpaulin, wood, iron, and mud) in southeast Delhi, Ajida, a Rohingya refugee, echoes Amal’s woes as she gently rubs her pregnant belly. Displaced from Myanmar because of ethnic persecution against her community, Ajida and her husband, a *maulana* (religious teacher), have only each other to depend on. She tells me (Raksha) and Huzara (my interpreter and a refugee herself) about her painful first trimester that had left her bedridden for days, unable to tend to her firstborn, manage her household, or even visit the hospital for treatment: “My husband had to manage everything, but he didn’t do it very well. The house was always messy, but he did take care of us.” Displacement has significantly affected their roles as parents and shifted care responsibilities. At present, their focus is on earning enough money to raise their family and finding a more permanent house outside the slum where they live. However, at times, Ajida begins to worry about the future. “Will my grandchildren also be refugees?” she wonders aloud.

For people enduring prolonged and forced displacement, social reproduction practices are crucial for survival amid limited access to employment, healthcare, education, and basic civil rights, as shown by Amal’s and Ajida’s stories. Social reproduction encompasses the daily practices that sustain life: biological reproduction, homemaking, caregiving, and maintaining relationships, culture, and community (Elias and Rai 2019). In this article, we engage further with the stories of refugees, probing the ways in which the everyday practices performed by forcibly displaced people to survive and reproduce can inform feminist theorizing on social reproduction. These practices are fraught with struggles against violent modes of capitalist accumulation and political marginalization and bring to the fore the multiple and complex economic, political, social, and environmental conflicts that characterize the current period in world politics.

In doing so, we align with feminists who conceive of all knowledges as situated and recognize the political nature of the everyday (Haraway 1988; Prügl and Tickner 2018), as well as with recent literature that learns from situated and experiential knowledges (Julian, Bliesemann de Guevara, and Redhead 2019; Poopuu 2022). Social reproduction scholarship has a longstanding tradition of challenging hegemonies and the binaries of production/reproduction and private/public. Nevertheless, this scholarship has remained relatively nation-centric, limiting understandings of how social reproduction is experienced in contexts of displacement, where citizenship rights are lacking. Echoing calls to pluralize feminist social reproduction approaches, specifically recognizing the heterogeneous lived experiences of people beyond Western contexts (Mezzadri et al. 2025), we argue that issues of (in)security are central to social reproduction. This can be a useful starting point to reflect on the gendered and racialized hierarchies that sustain the violences of prolonged displacement among refugees. While these experiences are context specific, highlighting them allows us to reflect on shared concerns that cut across different identity categories and national borders and to address the challenges to life-making processes among displaced communities.

Drawing on the experiences of Syrian refugees in Turkey and Rohingya refugees in India, this article aims to answer two questions: how is social reproduction experienced by people who have been forcibly displaced, and how does centering on their everyday practices of survival help us to engage with theories of social reproduction? Our main argument is two-fold. First, we show that for people living in conditions of prolonged displacement, social reproduction becomes a matter of survival that entails “making secure,” which involves reproducing a sense of security and a home. Thus, the practices of social reproduction engender security amid the insecurity of displacement. Second, they emphasize the role of not only the welfare state but also the security state and other non-state actors, which hinder social reproduction as much as they support it. We explore these dynamics through the concept of the “(in)securitization of social reproductive capacities,” highlighting the interplay between refugees’ endeavors to attain security and the securitizing efforts undertaken by state and non-state actors, which shape their access to basic services and opportunities. These include a range of practices that do not constitute social reproduction per se but that facilitate and support it. Our analyses foreground how refugees’ practices to make themselves and their families secure can, at times, actually increase their insecurity by exposing them to the violences of the security state. In this manner, the contradictory logics of (in)security are evident in everyday social reproduction.

In line with feminist security studies, we view the everyday as the site where “insecurities are felt, remembered, feared, negotiated and experienced” (Hedström 2021, 375). Thus, forced displacement is a lived experience navigated in everyday life, where agency is found not in resistance but in resilience – through the daily processes of negotiating and making sense of violence (Hedström 2021). Specifically, we illustrate how considerations of (in)security are central to the organization and performance of everyday social reproductive practices among refugees and vice versa. In doing so, we contribute to recent debates in feminist international relations to contend with the false binaries between feminist security studies (FSS) and feminist international political economy (FIPE) to recognize and challenge the interrelated gendered power relations that structure both the nation-state and the capitalist economy (Chisholm and Stachowitsch 2017; Elias 2015). While gender is a key axis of analysis in this article, it is entangled with issues of race and racialization. Here, we understand it in connection with refugees’ status as non-citizens, which is an important dimension of their marginalization. This also allows us to acknowledge the messy empirical realities of refugees’ lives, revealing the intersections of gender and race.

Further, we contribute to the existing debates within critical migration literature, which recognize the persistence of agency among refugees even in conditions of exclusion, oppression, and criminalization, particularly through acts of care, solidarity, and activism (Lisle and Johnson 2019; Moulin and Nyers 2007; Olivius 2017). Research on refugee agency among Syrian and Rohingya refugees in Turkey and India, respectively, brings to light the gendered and culture-specific manifestations of agency in displacement, for instance, through religious coping strategies, creating everyday routines for their families, homemaking, and building and maintaining neighborhood ties (Field, Pandit, and Rajdev 2023; Kanal and Rottmann 2021; Sezginalp Özçetin and Rottmann 2022). We center the practices of social reproduction performed by refugees to further challenge the representation of refugees as “bare lives” (Agamben 1998), and argue that refugees must negotiate their agency against the (in)securities of everyday life.

In the next section, we discuss the theoretical debates around survival and social reproduction, particularly in conditions of prolonged displacement. This is followed by a section in which we set out the empirical context of our analysis and describe our methods. Next, we examine everyday practices of survival and the ways in which they pluralize social reproduction feminism, focusing on three key themes: practices of making secure, the home, and the role of state and non-state actors. In conclusion, we discuss the implications of our findings for theorizing social reproduction within displaced communities in different contexts from the ground up.

## Forced displacement, survival, and social reproduction

At the end of 2022, the United Nations High Commission for Refugees (UNHCR) recorded 108.4 million forcibly displaced people worldwide (UNHCR 2023). Displaced populations are frequently victims of various types of violence, including physical, psychological, verbal, sexual, discursive, and harm to citizenship entitlements in their origin and destination countries. This violence is pervasive across time and space and is intensified by the pre-existing and interlocking racialized, gendered, and class-based hierarchies (Elias and Rai 2019). The violence characteristic of prolonged displacement is linked to power dynamics within the modern nation-state and global capitalist systems (Gill and Bakker 2003; Valiavicharska 2020). These linkages are particularly evident in how states mediate and sustain gendered social relations that undervalue or render invisible social reproductive work and in how states themselves have been transformed through decades of global economic restructuring (Elias and Rai 2019).

The emerging trends in forced displacement within and across borders are part of a larger process of the (in)securitization of migration. Increased surveillance, unlawful detention, and hostile border control measures discursively construct refugees as threats to the survival of state sovereignty, national security, and economic stability. This structural violence deepens the divide between citizens and non-citizens, further marginalizing already disenfranchised populations. Migrants, especially women, are often relegated to precarious labor with limited access to welfare services (Frydenlund 2023). The securitization of migrants thus insecuritizes non-citizens, confining them to the margins of labor markets and economies.

In the aftermath of forced displacement, the social reproduction of refugees is frequently challenged as a result of land dispossession, transfers, expropriation, and denial of social reproductive infrastructure (such as water, electricity, housing, protection, food, and healthcare) within destination countries. At times, it is also the result of deliberate state strategies to disrupt or damage the social reproductive capacities of specific communities (Lingham and Johnston 2024). This often leads to a situation where individuals are barely able to maintain a minimum level of subsistence (Whitener 2020). In this article, we further argue that this coercive control of social reproduction by states insecuritizes the survival of displaced populations. Thus, we acknowledge the direct links between social reproduction, (in)security, and survival.

Further, even though women are predominantly responsible for rebuilding communities, the non-recognition of their roles leads to their exclusion from accessing resources and decision making, thus reproducing unequal gender relations (Blomqvist, Olivius, and Hedström 2021). This non-recognition results in a specific form of gendered harm, or depletion (Rai, Hoskyns, and Thomas 2014). This form of gendered depletion is not simply the result of the continued burden of social reproduction; rather, women’s resources are already exhausted due to the multiple effects of conflicts leading to intensified depletion (Lingham and Johnston 2024). Depletion occurs at the individual, household, and community levels due to the unsustainable nature of social reproductive activities, especially when access to resources is inadequate. Understanding this depletion requires addressing power relations and the everyday negotiation of (in)security, particularly within refugee communities, focusing on processes rather than outcomes.

Therefore, we emphasize the ways in which considerations of (in)security are implicated in everyday practices of social reproduction performed by displaced women. In doing so, we are in conversation with FSS scholars who have illustrated that women’s gendered labor is significant in negotiating violence and (in)security and generating politics through the everyday (Hedström and Olivius 2020). These practices become a means through which they negotiate not only physical security, but a security of the self for themselves and their families in the context of their prolonged displacement and the impossibility of returning to their countries of origin (Rottmann and Nimer 2021).

In this article, we sit with, reflect, and think through the experiences of forcibly displaced Rohingya and Syrian refugees in India and Turkey, respectively, and what these mean for knowledge and sensemaking around social reproduction. We acknowledge the difficulties in bringing together two vastly different contexts where Rohingya and Syrian refugees have their own histories, distinct migratory trajectories, and experiences of displacement and marginalization. At the same time, we recognize the existence of political commonalities in their struggles to survive and secure their social reproduction. We argue that centering their everyday experiences of (in)security can contribute to pluralizing understandings of social reproduction and fostering conversations across contexts and disciplinary boundaries (Mezzadri et al. 2025). It allows us to theorize shared concerns that cut across different identity categories and national borders to recognize the challenges to life-making processes among displaced communities worldwide. Crucially, both contexts also help us to move beyond Western understandings of social reproduction as we interrogate the practices that constitute it from the ground up.

## Context and methods

This article explores the experiences of Rohingya and Syrian refugees in India and Turkey, respectively. Their experiences are illustrative of the violence inherent in the interlocking global capitalist and nation-state systems that marginalize refugees (Sassen 2016). The Rohingyas, stateless and persecuted in Myanmar’s Rakhine province, have faced ethnic persecution, land grabbing, and loss of political rights due to the 1982 Burma Citizenship Law (Haque 2017). Since 2011, many have fled to various parts of India, living in temporary settlements. Even though there are over 40,000 Rohingya refugees in India, the Indian state does not recognize their UNHCR-issued identification cards, which restricts their access to social services, employment, education, and healthcare.^4^ Meanwhile, Syrian refugees fled their homes due to the civil war in Syria, which started in 2011 following violent government repression of public mobilization and dissent. In Turkey, they predominantly live in the cities in the southeast and the surrounding rural areas, and in İstanbul. The Turkish Temporary Protection Regulation (TPR) grants Syrians limited rights without fully recognizing them as refugees, creating prolonged uncertainty for around 3.6 million Syrians (Adalı and Türkyılmaz 2020). This regulation restricts access to basic services and citizenship (Biner and Biner 2021).^5^

Both India and Turkey illustrate the everyday violence experienced by forcibly displaced communities. Both countries lack adequate national and international instruments to ensure the full protection of refugees. India is not a signatory to the Refugee Convention of 1951 and Turkey is a signatory of the convention but with a geographical limitation to Europe, which excludes Syrian refugees. Additionally, the irresolution of conflicts in Myanmar and Syria and the difficulties in the integration of Rohingya and Syrian refugees in destination countries suggest that their displacement will be prolonged for the foreseeable future. Another important dimension of their experiences is religion. Both communities are predominantly Muslim, but this plays out very differently in each context. In Turkey, shared Muslim beliefs are, at times, invoked by Syrian refugees and by the Adalet ve Kalkınma Partisi (Justice and Development Party) in debates around inclusion. Meanwhile, in India, Rohingya refugees’ Muslim identity is one of the primary reasons driving their exclusion, which has become more pronounced since the rise of Hindu nationalism in recent years. Though the intersection of religion with gender and race is vital to understanding the experiences of refugees, we have chosen to focus on the gendered and racialized nature of their insecuritization. This is an important avenue for future research to further pluralize social reproduction.

Our analysis draws on ethnographic research from two doctoral projects, one examining the politics of motherhood among Rohingya refugees in India and the other exploring the politics of social reproduction among informal laborers in Turkey. This article emerged from several years of collaboration and exchange of insights from our respective conversations and experiences navigating the complex and layered realities of refugees in India and Turkey. The data includes individual and group interviews, informal conversations, and participant observations (such as home visits and non-governmental organization (NGO) and community meetings) with refugee families and households, various civil society representatives, and community leaders, conducted over multiple visits to field sites in İstanbul and Şanlıurfa, Turkey, and in New Delhi and Haryana, India, between 2022 and 2024.^6^ We chose these field sites to incorporate the lived experiences of displacement and varying migration trajectories in rural, urban, and peri-urban settings. These are also the locations with a significant number of the Syrian and Rohingya populations in the two countries.

Our approach to this research recognizes the need for qualitative methodologies that center the experiences of migrants and refugees, thereby challenging dominant discourses in international politics, which often silence marginalized perspectives (Squire 2020). As female researchers, one from Italy researching with Syrian refugees in Turkey (Luisa) and one from India researching with Rohingya refugees in India (Raksha), both based at a European institution, the power dynamics inherent in this research are inescapable. Given that the interviews and participant observations were conducted with marginalized individuals navigating varied literacy levels, economic precarity, and a lack of citizenship, the interactions were inevitably influenced by layers of power asymmetries and colonial legacies. To safeguard the voluntariness of participation while protecting participants’ identities, informed consent was orally obtained and repeatedly negotiated throughout the research process. While recognizing our positionalities, we aim to center the narratives of marginalized communities and emphasize the co-production of knowledge (Johnson 2016). This involved ongoing open conversations with participants, treating them as experts of their experiences (Taha 2022), taking precautions to avoid re-traumatization,^7^ collaborating with local partners such as NGOs, and practicing an ethic of care toward the research participants.

More specifically, we draw on interviews with 21 Syrian refugees living in Esenyurt and Zaytinburnu (İstanbul) and Akçakale, Harran, and Eyyübiye (Şanlıurfa) and 40 Rohingya refugees living in southeast Delhi and Mewat (Haryana). The interviews were conducted at the participants’ homes, cafés, or NGO centers. All of the refugee participants were between 20 and 50 years of age and had received some form of primary education in their childhood. Though we prioritized women’s experiences during our data collection process, we aimed to achieve diversity regarding age, marital status, and, to some extent, gender. The majority of research participants were married, except for 16, who were either unmarried or widowed at the time of the interviews. Those who were or had been married had an average of four children, many of whom had been born in displacement.

We relied on personal connections and snowballing to get in touch with participants relevant to our sample and gradually built trust with our participants over time through repeated encounters. All of the interviews were conducted in Hindi or Rohingya (India) and Turkish, Arabic, or Kurdish (Turkey) and transliterated and translated into English with the invaluable help and insights of remunerated interpreters and research assistants. Interviews in both cases mostly revolved around means of subsistence since arrival in destination countries, challenges faced in accessing healthcare, employment, and education, division of labor in the household, activities that refugees and migrants engage in daily, time use, and ease of mobility and movement in the settlement and other areas.

To support our interviews and foster trust between ourselves and the research participants, we employed interpreters and assistants from refugee communities. Throughout the research process, we continuously reflected on how this affected power imbalances alongside our interpreters and involved them in our decision making, which ultimately facilitated meaningful exchanges. Thus, our analyses are the products of the continuous exchange among participants, interpreters, and ourselves. We recognize the crucial role of interpreters in facilitating our research. Instead of erasing their contribution, we make it visible in the ethnographic accounts that open our article. However, we chose to use pseudonyms to protect them from potential harm stemming from the sensitive contexts in which they live. While we acknowledge the extractive tendencies of ethnographic research (Abedi Dunia, Toppo, and Vincent 2023; Krystalli 2022), particularly in cross-cultural contexts, our practices allowed us to move toward a more participatory approach.

By reading together the materials collected from contexts that differ in significant ways, we do not aim to provide a comparative analysis. We also acknowledge that refugees are not a homogeneous category. Displacement is a complex social process, and Rohingya and Syrian refugees have rich, intersectional, and multilayered histories and identities. While recognizing that all knowledge is situated in the particular contexts of the researcher and the researched (Haraway 1988), we draw on commonalities in the lived realities of forcibly displaced populations across national borders to pluralize our understanding of social reproduction (Mezzadri et al. 2025). This helps us to highlight the common concerns of (in)security that refugees face in different contexts against the backdrop of the exclusionary politics of nation-states. We emphasize how (in)security challenges, disrupts, and erodes even the most intimate aspects of everyday life among displaced communities. By focusing on these concepts, we can foster conversations across different contexts, respecting the varied experiences of these insecurities while embracing a shared human struggle for survival.

## Expanding the scope of social reproduction feminism

In this section, we engage with the existing debates on social reproduction by analyzing the everyday practices performed by Rohingya and Syrian refugees in India and Turkey, respectively, in three ways. First, we highlight the significant increase in care responsibilities and housework, and the role that these practices play in making secure – that is, maintaining a sense of security for the self, the household, and the community amid forced and prolonged displacement. Second, we analyze the home as a more-than-private space of politics where (in)security is constructed, reproduced, and challenged. Third, we examine the practices that refugees perform to negotiate insecurity in their daily lives. We propose the concept of the (in)securitization of social reproductive capacities to more fully integrate matters of insecurity within social reproduction theorizing. Further, we highlight supplementary practices, such as avoiding security officials and checkpoints, commuting, and creating and maintaining relationships with state and non-state actors, performed by refugees to facilitate social reproduction. In bringing these dynamics to light, we demonstrate that these practices are central to social reproduction in a way that challenges the false binaries between FSS and FIPE.

### “Making secure” through the practices of social reproduction

Forced displacement significantly impacts the care responsibilities of the people affected, particularly those of women. Nevertheless, even in the aftermath of violent displacement, people continue to practice different forms of care and maintain social relationships (Krystalli and Schulz 2022). In contexts of violence, the practices of social reproduction that refugees perform become a matter of survival that entails creating and maintaining a sense of security for the self, for the household, and for the community amid the insecurity of displacement (making secure). These practices are relational and embedded in transnational relations of gendered and racialized power that extend beyond the household. These practices travel across borders with refugees themselves and are not static; on the contrary, they are constantly renegotiated in response to the everyday realities of displacement.

Our conversations with refugees in India and Turkey bring to light the centrality of a range of practices of social reproduction and their persistence in the lives of women. For instance, when asked about the role played by women in the aftermath of displacement, a Rohingya woman responded:

What does a woman do? This is what we do: we cook, we clean, we take care of the house, we take our children to school, we cook again, we sleep. In our community, women stay at home – they don’t go out to work unless there is a compulsion. (interview, female Rohingya refugee, November 23, 2023)

As this excerpt demonstrates, though social reproduction practices – especially those involving care and housework – help to achieve a sense of normalcy and nurture family and community amid displacement, they are embedded in gendered expectations within the community that reproduce displaced women’s insecurities. Her words illustrate the importance of routine and repetition, and the need to not only maintain the physical space but also nurture and ensure the well-being of family members. At the same time, her mention that “women stay at home” and usually do not go out to work speaks to social expectations and norms regarding women’s roles within the community. These practices provide stability, but also intensify women’s exclusion and patriarchal subordination by restricting women to their homes. This exclusion is compounded by their racialized status as “illegal migrants” in India, which exposes refugees to violence and discrimination. As explained by a project coordinator of an NGO, “[m]any of the women refuse to participate and come alone to our NGO center for the fear of running into the police or fear of harassment in public spaces” (field note, female Indian NGO official, October 9, 2024). Their illegal status results in exclusion from the labor market and reinforces women’s confinement to the home. In the words of one interviewee, “[m]any of us women, we want to work. We want to go outside our homes, but there are no jobs available for us” (interview, female Rohingya refugee, October 4, 2024).

Our interviews resonate with this ambivalent conceptualization of social reproduction practices both as reproducing gendered oppression – which is exacerbated by forced displacement and racialized exclusions – and at the same time as expressions of agency evident from the interviewee’s insistence on “want[ing] to work” and women’s conscious decision to remain at home to avoid harassment and the fear of violence outside the home. In fact, refugee women are mainly responsible for taking care of their children and their homes. Their days are divided between cooking, cleaning, taking care of children and the elderly, dropping children off and picking them up from school, and shopping for food.

These practices of social reproduction are crucial to making secure, but they are also depleting for women as they confront the loss of family and community. The traumatic effects of displacement have significant negative effects on the continued performance of various social reproductive practices. In turn, this impacts refugees’ sense of self, including in relation to children and their roles as mothers. As described by Amal, the Syrian refugee whose story opened this article:

I am sick and constantly feel suffocated and weak. I’m afraid of losing my children. I feel like I’m possessed […] Sometimes, I tremble uncontrollably, and at times I can’t focus on my children or household duties. I wasn’t like this before I had this illness […] This happened after a great sadness. I had two brothers, and they passed away [during the war], and I mourned them greatly. The thing that saddens me the most is that I can’t take care of my children as much as I want to or fulfill their requests. […] My relationship with them is very good, but now, since my illness, I feel like I’m distant from them. I am very sad because I feel like I can’t help them or fulfill their requests. I’m afraid of losing them and them losing me because the most important thing in this life is the mother, and I don’t want them to be deprived of me. (interview, female Syrian refugee, June 21, 2023)

This excerpt illustrates the emotional effort involved in fulfilling social reproductive responsibilities and the ways in which making sense of trauma and loss unfolds relationally in and through these practices. Her statement indicates a significant disruption in her security, caused by the loss of her two brothers during the war and her subsequent mourning. While, as she describes, the most important aspect of life is motherhood, she finds herself unable to meet her children’s needs. This inability demonstrates the challenges that forcibly displaced people, and women in particular, face in performing social reproductive practices. At the same time, her fear of losing her children and the reciprocal fear of them losing her underscores the centrality of relationships and relationality to security. Her words demonstrate the connections between the need for safety, social reproduction, and (in)security. They highlight how personal traumas and disruptions as a result of violent conflict can profoundly affect an individual’s sense of self and well-being, especially in the context of caregiving roles such as motherhood. The fragmentation of the self and related sense of loss – or embodied “illness,” in the words of Amal – is a crucial dimension of (in)security and a relatively underexplored theme in feminist social reproduction scholarship. Addressing memory and trauma can deepen this scholarship by revealing new narratives not only of war but also of the material struggles for survival experienced by marginalized communities (see for example Shringarpure and Cantelli 2023).

In addition, practices of social reproduction are intergenerational; they involve children’s futures, as displaced people, and women in particular, reconfigure transnational spaces to make sense of themselves and a home for their communities. Indeed, our interviews and observations illustrate that these practices are not only about fulfilling requests and meeting gendered duties; on the contrary, they also involve consciously helping children to come to terms with experiences of violence, exploitation, and resettlement (Al-Dabbagh 2022). In the words of a Rohingya refugee:

When I am unable to protect my children, I feel like I have failed as a mother. I want to provide for them, feed them, and educate them. I always remind them to not leave their studies, to be strong, and everything will be OK. (interview, female Rohingya refugee, May 17, 2023)

Her efforts to protect, provide for, and guide her children contribute to the creation of a supportive environment in which they feel secure. Crucially, practices of social reproduction involve a desire and commitment not only to fulfill basic needs but also to contribute to the education and overall development of her children, reflecting a broader orientation to the future. Put differently, though these social reproductive practices are embedded in intersectionally gendered power relations and related forms of oppression, their value is significant in the sense that they have social meaning, represent a way of practicing motherhood, and assert agency and belonging for oneself and one’s family, thus maintaining security and hope for the future amid the uncertainty and insecurity of displacement. A focus on social reproduction thus centers the perspectives of the displaced in discussions of security, which usually prioritize the state, while pushing FIPE to make visible and reckon with these issues.

### The home as a more-than-private space of politics

The home, as a site of circulation of labor and capital, is central to theories of social reproduction. It is often viewed as a “private place” where the essential physical and embodied labors of making and raising babies, securing food, clothing, shelter, healthcare, safety, and education, and maintaining relationships are performed along gendered, racialized, classed, and locational axes. However, we argue that the home is also a political space where practices and ideas about belonging, insecurity, and exclusion are socially reproduced by refugees themselves and, increasingly, the state and other non-state actors (Clark 2016). The reconstruction of the home as a more-than-private space is made possible by the increasing preoccupation of modern nation-states with indexing the everyday lives of people, including refugees, within their borders as a matter of promoting security and sustaining certain kinds of life (those of citizens) while (in)securitizing those of others (those of non-citizens).

This insecuritization of everyday life is facilitated by various contemporary security practices that depend not only on force and violence but also on significant interventions by state and non-state actors in the social reproductive practices performed within the homes of people such as the forcibly displaced Syrian and Rohingya refugees we interviewed. On the one hand, this is carried out through socio-economic development programs such as those for education, healthcare, fertility, and family welfare; on the other hand, it includes coercive social control or neglect of the social reproductive capacities of undervalued others through securitizing practices.

**Figure 1.**
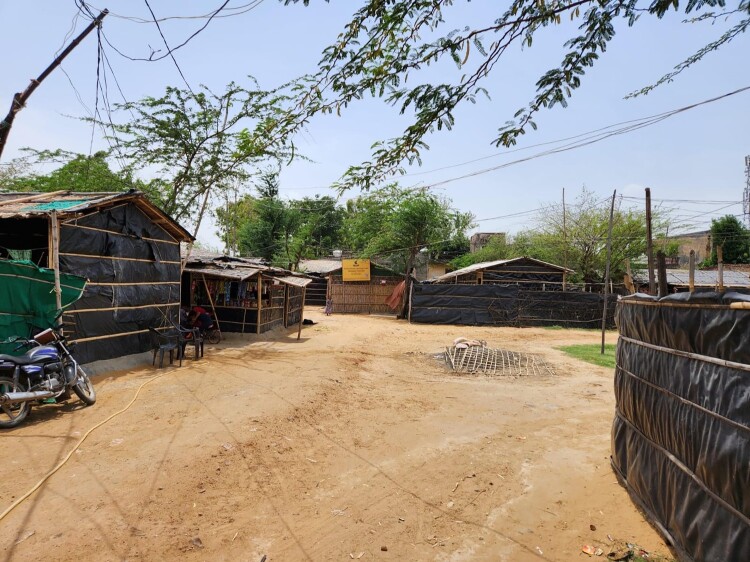
Homes within a Rohingya refugee settlement in India.

**Figure 2.**
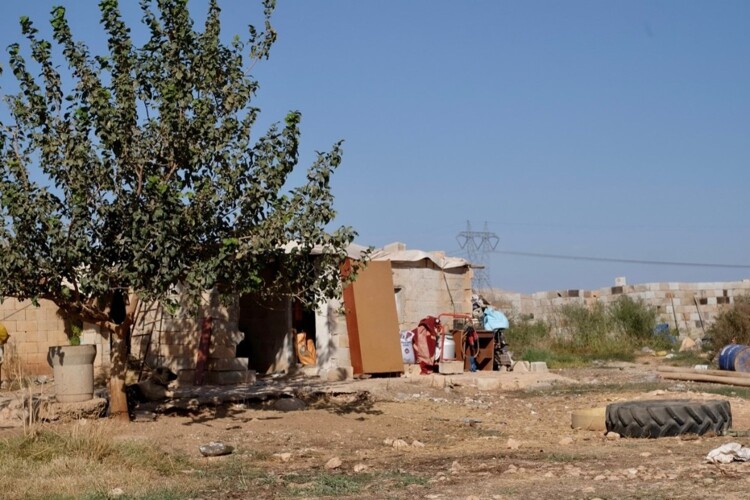
Home of a Syrian refugee family in Turkey.

In the everyday lives of forcibly displaced refugees, the home, as portrayed in Figures 1 and 2, is a place where several state and non-actors (such as the police, local government staff, officials from the UNHCR for the Rohingyas in India or the Red Crescent for Syrian refugees in Turkey, and other civil society organizations) intervene in various ways, such as through surveillance, patrolling, family surveys, and community meetings. The home becomes a political space where security – and the consequent insecurity – is felt, constructed, and renegotiated continuously between these various actors and refugees through routine interactions. The following words of a Rohingya refugee in India are emblematic of these routine interactions:

[A]t least twice a night, there is a police patrol here. Every day! They make rounds once, and sometimes they make rounds three times. They check if the security is strong. No one should stay outside; everyone should stay in their shanties. (interview, female Rohingya refugee, October 17, 2023)

The regularity of patrols by the police, as this quote shows, is a way of maintaining “security” as understood by the state through continuous surveillance of the space where the refugees reside. The home is subject to external scrutiny and monitoring. State actors, such as the police in this case, actively shape and regulate the behaviors and movements of those within the refugee community, redefining the boundaries of their homes. These practices illustrate the tension between “security” as understood by the state and as experienced by the refugees.

Similarly, in Turkey, some refugees from Syria voiced concerns over randomized surveillance by non-state actors, such as transnational and national NGOs, which are integral to the security apparatus of modern nation-states (Gupta and Ferguson 2002). As one interviewee put it, “[y]ou need an address and place of residence […] [I]f they come and don’t find the [applicants], they cut off the support” (interview, male Syrian refugee, June 21, 2023).

Thus, distinctions of private and public – a key gendered binary questioned in the social reproduction literature – are further blurred by the continuous movement of state and non-state actors into the home, typically conceived as “feminine,” and surrounding spaces (such as the camp). Often, these measures of coercive control by state and non-state actors generate fear and jeopardize the security of displaced communities, especially by interrupting their ability to engage in social reproductive activities. Routine surveillance means that, at times, individuals and families find it challenging to perform their everyday social reproductive practices efficiently. For instance, in the Rohingya settlements in New Delhi, when single mothers are summoned by the police or the UNHCR for administrative tasks such as registering their family members or collecting monthly aid cheques, they are often forced to leave their infant children alone at home, disrupting daily routines, including cooking, cleaning, caring for their children and sending them to school.

Additionally, the home is also a space where practices and ideas around reproduction, motherhood, marriage, and family are discussed and materialized (Clark 2016). Far from being private realms of everyday life, in India and Turkey, there is a significant presence of non-state actors in conversations around these ideas with displaced communities. For instance, in the Rohingya refugee settlements in New Delhi, family sizes are often perceived as too large by NGOs. Often, reproductive choices, particularly those of women, are called into question in NGO-led community meetings and door-to-door awareness campaigns in their homes. Thus, the home is also the place where gendered and racialized identities are negotiated between refugees and other actors.

In Turkey, while conducting routine home visits to verify eligibility for financial support, NGO officials identify large family sizes among Syrian refugees as a key source of insecurity. An NGO employee explained:

Some families have ten children under 18. They need support […] If Syrian women don’t have more children, they might fear that their husbands will marry more wives. They also want to go back home after the war and have a big family to rebuild their community. (field note, female Turkish NGO official, June 17, 2023)

While the desire for larger families might be seen as a way to rebuild communities after displacement, it is also subject to external scrutiny. At times, these interventions are viewed through a racialized lens. This discussion reflects how social reproduction is tied to generalized assumptions about refugee communities and is viewed as a source of cultural difference (such as polygamy in the excerpt above) and vulnerability. In this manner, social reproduction is linked to questions of (in)security, exclusion, and gendered and racialized hierarchies within destination states.

### Negotiating the (in)securitization of social reproductive capacities by state and non-state actors

Centering the experiences of those who have been forcibly displaced brings to the fore the multiple ways in which not only the welfare state but also the security state is implicated in social reproduction. As our discussion above has revealed, (in)security is created, managed, and challenged in various ways in the context of displacement. Arguably, insecurity also results from neglect and, at times, deliberate attacks on the social reproductive capacities of displaced communities. These include the lack of adequate housing facilities such as refugee camps (for Rohingya refugees in India) and precarious rental homes or tents (for the majority of Syrian refugees in Turkey) as those shown in the previous section, as well as forced displacements from one settlement to another, and sporadic access to electricity and water, which directly impact the everyday survival of these communities (Whitener 2020). This form of violence, which we refer to as the (in)securitization of social reproductive capacities, further excludes and marginalizes displaced communities within destination states and also creates conditions for their depletion, as we have seen in the previous sections.

While state and non-state actors equally (in)securitize and police social reproduction, the practices through which refugees survive and continue to reproduce life are also significant. Refugees actively confront security practices through tactics such as making themselves visible to the state through registrations, or making themselves invisible through avoidance, maintaining social ties with state and non-state actors, and commuting, highlighting the role played by everyday negotiations against insecurity, such as threats of detentions, arrests, deportations, and surveillance.

To survive, members of forcibly displaced communities need to continuously negotiate the boundaries of legality/illegality and visibility/invisibility to secure social reproduction for their families and communities. Procuring some form of identification papers is of utmost priority for the forcibly displaced, who often arrive in destination countries without legal documentation. In India, the UNHCR is primarily responsible for the provision of this documentation. However, in the absence of formal recognition by the Indian state, refugees in possession of UNHCR asylum cards exist in the gray area between legality and illegality. Ensuring that their identification documents are up to date and legitimate is the responsibility of refugees. Often, this can be a source of stress. For instance, in a conversation with a female Rohingya refugee in Delhi, she revealed that in 2021 her refugee card was deactivated by the UNHCR without any prior notice:

I called the UNHCR many times, but they did not answer. My card stopped working last year and for seven months I lived in fear of detention and the police […] For seven months, I did not go anywhere for fear of the police. (interview, female Rohingya refugee, May 17, 2023)

As this excerpt suggests, everyday life was punctured by the fear of being apprehended by the police, which prevented our interlocutor from engaging in daily activities outside the refugee camp. In particular, she would avoid going to the hospital, taking her children to school, or even fetching amenities as basic and essential as water because of the fear of being caught. Thus, to avoid the risk of detention and deportation, at times, refugees restrict their mobility beyond the settlement. This severely impacts their abilities to take up employment and care for themselves and their families, thus directly impacting everyday social reproduction. In this manner, capacities and opportunities for social reproduction are restricted – at times, deliberately – and create experiences of insecurity and exclusion among refugees.

One way in which Rohingya refugees navigate this insecuritization is by forming social bonds with local policemen, humanitarian agencies, and lawyers. Some of them volunteer as community leaders and mobilizers with local NGOs and act as key intermediaries and informants relaying important information about births, deaths, detentions, and deportations. The following quote from a male community leader in a Rohingya settlement is illustrative of the role that refugees play in the community:

The position of a community leader was created so that individuals like me, representing around 100 to 150 people from our community, could manage and discuss issues collectively. We gather input from everyone, discuss matters that come from higher authorities, and make decisions after thorough discussions. Not everyone can directly communicate with the authorities, so I play a role in relaying the community’s needs and concerns. This collective decision-making process strengthens the community and helps in conveying accurate information to the authorities, such as the UN, regarding our current needs, especially during specific weather conditions like the rainy season. (interview, male community leader in Rohingya settlement, October 28, 2023)

Alternatively, some Rohingya refugees try to acquire fake national identification cards that identify them as Indian nationals. This enables them to access employment opportunities, rations, and subsidies only available to Indian citizens. Thus, these practices of registration, avoidance, forming social bonds, and procuring fictitious identification cards, though not practices of social reproduction on their own, allow refugees to negotiate some form of security of the self in the face of the vulnerability and risks associated with prolonged displacement. By extension, these practices also allow forcibly displaced people to secure and sustain social reproduction by negotiating insecurities that act as barriers to their survival. Recognizing these negotiations has important implications for social reproduction theories. They point to the role of the security state and other non-state actors in hindering social reproduction (such as by not renewing cards), and to the existence of collaborative strategies within the community that cannot be reduced to the category of individual, feminized labor and yet play a central role in social reproduction. Collective efforts to maintain life thus contend with and resist the xenophobic othering and racialization of migrant communities perpetrated by states and their apparatuses of repression, control, assimilation, and erasure (Valiavicharska 2020).

This (in)securitization of social reproductive capacities can have indirect consequences in other aspects of refugees’ lives. For instance, as a result of not possessing the right identification cards, daily mundane activities such as commuting can expose refugees to heightened risks and become more time consuming, stretching the length of the working day with negative effects on refugees’ well-being. Commuting is an essential activity of social reproduction as it involves traveling for reasons related to meeting basic needs, including purchasing food, accessing services such as education and healthcare, and labor opportunities. For Syrian refugees, it can become a particularly dangerous endeavor if they do not possess an identification card issued in the place in which they are registered as “temporary subjects,” which is a common problem due to the bureaucratic barriers and costs involved in transferring residence under the TPR. As described by one interviewee in İstanbul:

My ID is not from İstanbul, it’s from Nevşehir. I came to Turkey late and I couldn’t settle. Once, the police caught me and told me to go back to [my] state and not leave. […] In the morning, I struggle with the transportation. I prefer to walk than us[e] the bus because I got pickpocketed twice. Once, my phone was stolen, and the second time, my wallet got stolen. You know how crowded the bus is. Once, my wallet with my papers were stolen. I came back later and found them thrown next to the driver, and the wallet was empty. There was no money. […] That’s why I prefer [to] walk, even though it takes me about an hour or 45 minutes. (interview, male Syrian refugee, November 19, 2022)

As the excerpt shows, commuting is a depleting activity that hinders the interviewee’s capacity for social reproduction. Instances of being pickpocketed and having personal belongings stolen, including the wallet with important papers, underline the vulnerability associated with not having the right identification card. This vulnerability is not merely a result of individual circumstances but is tied to racialized constructions of citizenship and nationhood that categorize some individuals as “types” that either do or do not belong (Sharma 2015). While female research participants from Syria expressed facing difficulties with commuting due to fears of discrimination in public spaces, these were exacerbated by the burden of domestic tasks and childcare at home, which constrains their mobility. Mundane activities such as commuting thus become emblematic of the struggles that forcibly displaced people face along the lines of gender, race, class, and citizenship status in their daily lives as they confront (in)securitization practices in their destination country.

Faced with insecurity and exclusion, our conversations with refugees reveal that they must innovate and strategize to survive and secure their social reproduction. These strategies can expose refugees to greater insecurities, revealing the contradictory logics of (in)security embedded in the everyday realities of prolonged displacement. In both the Indian and Turkish contexts, refugees are dependent on sporadic cash flows from international agencies and NGOs. Ensuring access to these forms of support requires significant efforts to maintain close ties with these organizations and keep track of changing eligibility requirements. Though these activities, separately or in conjunction, are not sufficient to secure social reproduction sustainably, they are illustrative of refugees' negotiations of states’ (in)securitization of social reproductive capacities.

Our findings demonstrate that continuously negotiating for survival and securing social reproduction in displacement implies navigating a complex set of state security measures every day. Refugees’ practices to secure social reproduction (discussed in the previous sections) can expose them to conditions of deeper insecurities. Therefore, security and insecurity are mutually constitutive processes with serious consequences for social reproduction. This also illustrates the involvement of not only the welfare state but also the security state in social reproduction.

## Conclusion

Amal and Ajida, whose stories opened this article, continue to live in displacement with their families. Our conversations with them have revealed the permanence of the insecurities that they experience every day and their constant struggle to survive. Bringing to light the everyday practices of survival that refugees perform to reproduce life in conditions of prolonged and forced displacement and affirming their importance for feminist theorizing on social reproduction has been at the heart of our analysis. Drawing on ethnographic research with Rohingya and Syrian refugees in India and Turkey, respectively, we have expanded the scope of social reproduction feminism to reflect on the mutually constitutive relationship between security and practices of social reproduction. This includes practices to maintain a sense of security (making secure) for the self, for the household, and for the community amid forced and prolonged displacement, as well as the home as a space of politics, and the (in)securitization of social reproductive capacities by state and non-state actors in contexts of displacement. These practices are overlapping and complementary. While displacement is characterized by multiple forms of violence, the everyday survival practices performed by refugees allow the enactment of constrained forms of agency and envisioning different futures.

Across different contexts, the evidence presented in this article has demonstrated that social reproduction is difficult for both Rohingya and Syrian refugees in their destination countries. Moreover, as a result of gendered expectations, lack of access to basic resources and documentation, surveillance, and racialization, it is further insecuritized. This insecuritization manifests in various forms across both contexts, but highlights the common struggle to survive among displaced communities. Building on previous feminist scholarship that has challenged the idea of refugees as “bare lives” (see for example Lisle and Johnson 2019; Olivius 2017), we have shown that forcibly displaced populations continuously negotiate social reproduction within their communities through everyday practices to survive, care, and maintain a sense of security, identity, and community despite scarce resources, outright hostility, and violence. The prolonged nature of the displacement that they experience further complicates the idea of survival, which is linked to the (in)securitization practices of nation-states. Through our discussions, we have also disrupted the binary of security/insecurity by illustrating their mutual constitution.

Our contribution to social reproduction debates is both theoretical and empirical. First, we have provided an empirical contribution in terms of attending to the experiences of Rohingya and Syrian refugees themselves. Second, while social reproduction definitions and analyses have proliferated in recent years, these have seldom taken into consideration the lived realities of displaced communities around the world, for whom social reproduction is increasingly challenging. By centering their experiences as a starting point for theory building, we have contributed to recent efforts to pluralize social reproduction (Mezzadri et al. 2025), and have provided a framework that holds together feminist security and political economy issues, thus bridging disciplinary divides. This approach fosters conversations across different contexts, recognizing the unique struggles of displaced communities as both significant and interconnected through their experiences of insecuritization by nation-states.

While we reject a single human condition of prolonged displacement, juxtaposing different experiences allows us to draw commonalities and highlight their political importance across national borders, as a way of fostering meaningful conversations among feminists doing research in contexts beyond the West. Following recent calls for thinking more deeply about ways to build connections across different empirical contexts (choi et al. 2023), we have foregrounded the intersection of social reproduction, (in)security, and survival as a starting point to interrogate the practices that constitute them from the ground up. Our article has been motivated by this goal – one that recognizes the heterogeneous, yet shared, struggles for survival and security of displaced individuals and communities across borders.

## Notes

1 Research participants and interpreters have been given pseudonyms to protect their identities.

2 In this interview, the research participant used the terms “war” and “great sadness” to describe the events happening in Syria and affecting her family. Another research participant made a distinction between the terms “revolution,” which referred to the Syrian people’s uprising against Bashar al-Assad's regime, and “war,” which signified the regime’s repression of dissent and escalation of violence.

3 This article was written before the fall of al-Assad’s regime on December 8, 2024. We acknowledge the uncertain and rapidly evolving circumstances currently faced by Syrian refugees and note that the findings presented here capture a specific historical moment.

4 For a historical and cultural background of the Rohingya community and their status in Myanmar, see Farzana (2017). For an analysis of their displacement in South Asia, see Chaudhury and Samaddar (2018). For a gendered analysis, see Akhter and Kusakabe (2014), Field, Pandit, and Rajdev (2023), and Frydenlund (2020). For a critical analysis of their lived experiences in India, see Mitra and Şahin-Mencütek (2024).

5 For a critical analysis of political and social realities in contemporary Turkey, including refugee policies and practices, see Jongerden (2021). For a gendered analysis of the Syrian refugee crisis, see Freedman, Kıvılcım, and Baklacıoğlu (2017). For analyses of Syrian refugees’ experiences in labor markets, see Dedeoğlu (2022), Pelek (2020), and Rottmann and Nimer (2021). For a critique of the public discourse around Syrian refugees, see Sert and Danış (2020).

6 This research has been approved by the ethics committee at the Geneva Graduate Institute where we are based. All of the interviews were conducted with the informed verbal consent of the participants, who had the opportunity to withdraw consent at any stage during the research.

7 These precautions included avoiding intrusive questions and paying attention to research participants’ body language, showing empathy, and letting them control the flow of the conversation.
